# Neglected tropical diseases in Brazilian children and adolescents: data analysis from 2009 to 2013

**DOI:** 10.1186/s40249-017-0369-0

**Published:** 2017-11-03

**Authors:** Eduardo Brandão, Sebastián Romero, Maria Almerice Lopes da Silva, Fred Luciano Neves Santos

**Affiliations:** 1 National Reference Service for Filariasis, Aggeu Magalhães Institute (Fiocruz-PE), Recife, Pernambuco Brazil; 2 Faculty of Medical Sciences, National University of Rosario, Rosario, Santa Fe Argentina; 3 Laboratory of Communicable Diseases, Parasitology Department, Aggeu Magalhães Institute (Fiocruz-PE), Recife, Pernambuco Brazil; 4 Laboratory of Pathology and Bio-Intervention, Gonçalo Moniz Institute (Fiocruz-BA), Salvador, Bahia Brazil

**Keywords:** Parasites, Visceral leishmaniasis, Malaria, Schistosomiasis, Chagas disease, Disease notification, Residence characteristics, Brazil

## Abstract

**Background:**

Neglected tropical diseases (NTDs) prevail in conditions of poverty and contribute to the maintenance of social inequality. Out of the NTDs prioritized by the Brazilian Ministry of Health, four parasitic infections require mandatory notification: acute Chagas disease, leishmaniasis, malaria, and schistosomiasis. Data on the behaviour of these NTDs in the young population are currently limited. This study seeks to analyse the epidemiological aspects of these parasitic infections in children and adolescents in Brazil.

**Methods:**

A retrospective exploratory ecological study was conducted. A spatial analysis of the cases reported between 2009 and 2013 in individuals aged between 0 and 19 years that were notified through the Health Notification Aggravation Information System (SINAN) was performed.

**Results:**

In total, 64,567 cases of cutaneous and visceral leishmaniasis, malaria, schistosomiasis, and acute Chagas disease were recorded in the SINAN database, representing a rate of 20.15 cases per 100,000 inhabitants. The average age of the cases was 12.2 years and 62.32% were male. Four hundred and three deaths related to these obligatorily reported parasites were recorded, indicating a case fatality rate of 0.62%. Visceral leishmaniasis and acute Chagas disease had the highest rates of lethality. A heterogeneous spatial distribution of the studied parasites was observed.

**Conclusions:**

The number of cases and the lethality rate described in this study show that these diseases still represent a serious problem for public health in Brazil. This points to the need to encourage new research and the reformulation of social, economic, and public health policies aimed at ensuring better health and living conditions for all individuals, especially those among the populations considered vulnerable, as is the case of the young.

**Electronic supplementary material:**

The online version of this article (10.1186/s40249-017-0369-0) contains supplementary material, which is available to authorized users.

## Multilingual abstracts

Please see Additional file 1 for translations of the abstract into the five official working languages of the United Nations.

## Background

Neglected tropical diseases (NTDs) comprise a large group of tropical infections that are strongly associated with poverty and concentrated mainly in slum and remote rural areas of developing regions in Africa, Asia, and the Americas [1]. These diseases are characterized by their high prevalence, chronicity, and disabling features [2]. The World Health Organization (WHO) recognizes 17 NTDs that blight the lives of a billion people in 149 countries and threaten the health of millions more [1].

In Brazil, nine NTDs affect the population, of which seven (dengue, acute Chagas disease, leishmaniasis, malaria, schistosomiasis, leprosy, and tuberculosis) entail obligatory notification and are considered priorities for prevention and control, owing to their severity and harmful socioeconomic consequences (Additional file 2) [3–5].

From a biological perspective, children and teenagers represent a group that is vulnerable to NTDs owing to malnutrition and impairment of cognitive development, which demands more attention from healthcare agencies [6, 7].

Leishmaniasis, in its various forms, has been reported in urban areas of Brazil [8–11]. It completely destroys the mucous membranes of the mouth and throat and leaves permanent scars, leading to massive suffering. Visceral leishmaniasis (VL) occurs in 12 countries in Latin America, with more than 95% of cases reported in Brazil [12]. This disease attacks internal organs and is fatal if neglected. The age of the infected individual influences the outcome of infection, as a high proportion of patients are children [13]. A study carried out in a pediatric referral hospital in Pernambuco showed a high mortality rate resulting from VL in children aged below 13 years [14].

Malaria, another disease of public health importance, is equally fatal if untreated. Pregnant women and children below the age of 5 years are the most vulnerable groups [15, 16].

*Schistosoma mansoni* infection is also of considerable public health importance in tropical countries, mainly affecting school-age children, women of childbearing age, and workers who are in frequent contact with contaminated fresh water [17]. The damage to the intestinal tissue results from the large numbers of eggs released by the flukes. If untreated, the disease can lead to long-term, irreversible health effects, including periportal fibrosis, liver cirrhosis, and obstruction resulting from the depositing of eggs in the small portal venules [18]. Children are especially vulnerable in endemic areas, where poor hygiene, lack of sanitation, and recreational water use prevail [19]. Severe schistosomiasis impairs cognitive development, disrupts school attendance, contributes to malnutrition in children, and causes poor growth, although the effects are usually reversible with treatment [20].

Chagas disease is a vector-borne disease responsible for 5.7–9.4 million cases in the continental Western Hemisphere [21, 22]. Every year, due to the disease, 14,000 deaths occur in 22 endemic countries in Latin America [23]. The epidemiological pattern of Chagas disease has changed because of the interruption of transmission following *Triatoma infestans* elimination in Brazil and other areas of Latin America [24]. Protracted infection can lead young adults to develop heart conditions that require hospitalization, thereby reducing the labour force [20].

Knowledge of the distribution of NTDs in endemic areas is fundamental to the follow-up and assessment of interventions and the effectiveness of control measures. Attention has recently turned to epidemiological studies considering the spatial distribution of NTDs in the general population [25–28]. Owing to the scarcity of information describing NTDs in Brazilian children and adolescents, the epidemiological aspects and distribution of visceral and cutaneous leishmaniasis, acute Chagas disease, malaria, and schistosomiasis in individuals aged up to 19 years reported in the Health Notification Aggravation Information System (SINAN) database from 2009 to 2013 were assessed.

## Methods

### Study area

**Fig. 1:**
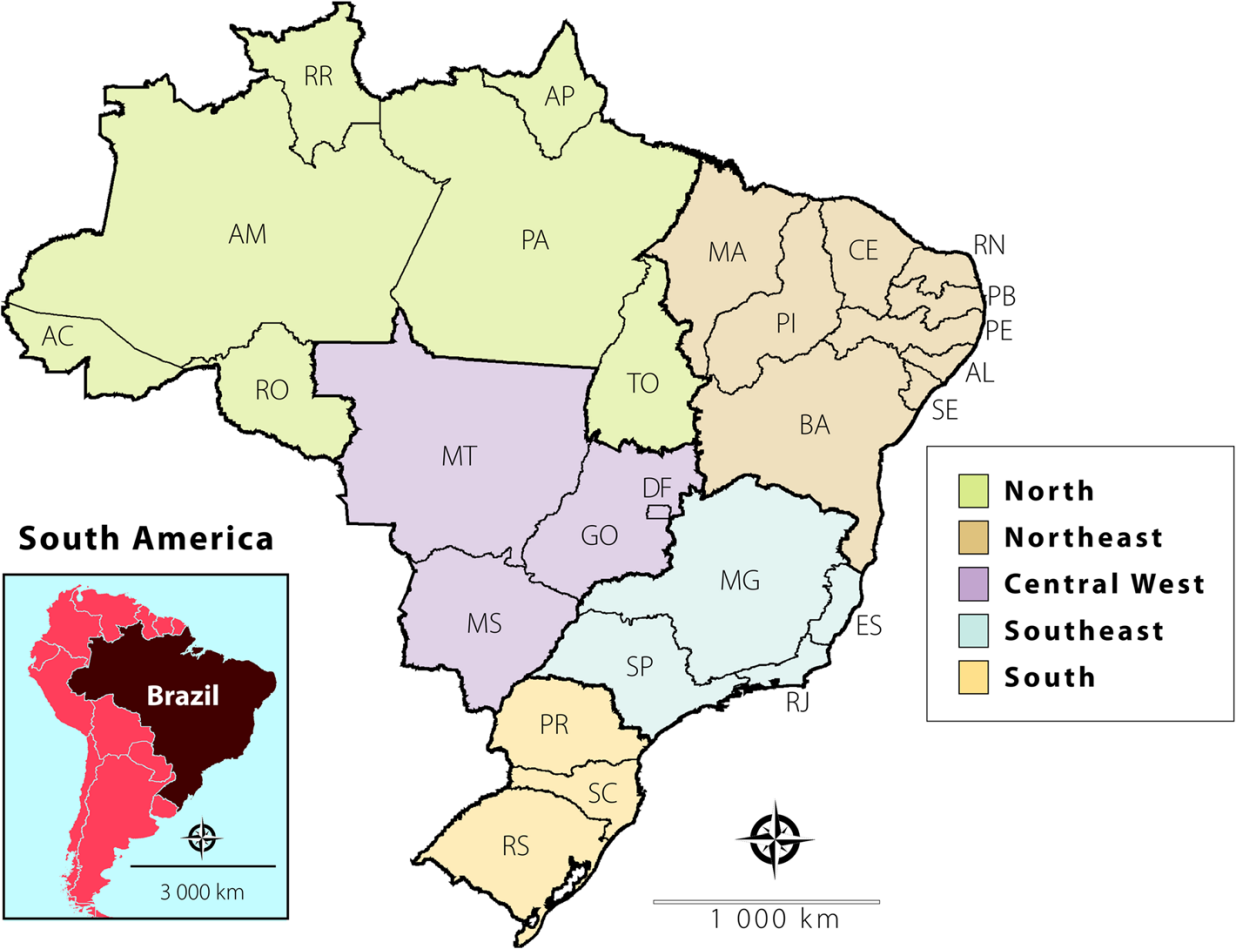
Political and administrative division of Brazil into five regions. North (AC: Acre, AM: Amazonas, AP: Amapá, RO: Rondônia, and RR: Roraima); Northeast (AL: Alagoas, BA: Bahia, CE: Ceará, MA: Maranhão, PB: Paraíba, PE: Pernambuco, PI: Piauí, RN: Rio Grande do Norte, and SE: Sergipe); Central West (DF: Distrito Federal, GO: Goiás, MS: Mato Grosso do Sul, and MT: Mato Grosso); Southeast (ES: Espírito Santo, MG: Minas Gerais, RJ: Rio de Janeiro, and SP: São Paulo); and South (PR: Paraná, RS: Rio Grande do Sul, and SC: Santa Catarina)

This study was conducted in Brazil, the largest country in Latin America and the fifth largest in the world, with a total area of 8,515,767 km^2^. The Brazilian Federation comprises 26 states and a federal district (containing the capital Brasília), which may be grouped into five regions: North, Northeast, Central West, Southeast, and South (see Fig. 1). According to the 2010 demographic census, the total population of Brazil was 190,755,799 inhabitants, 32.99% of whom were children or adolescents aged up to 19 years (available at http://www.ibge.gov.br).

### Study design

An ecological retrospective study was conducted using data obtained from the open-access SINAN database, taking into account reported cases of cutaneous and visceral leishmaniasis, malaria, schistosomiasis, and acute Chagas disease in each notification microregion from 2009 to 2013 (available at http://datasus.saude.gov.br). All reported cases aged between 0 and 19 years were included in this study.

The SINAN database provides information for the creation of indicators for monitoring the distribution of certain diseases in Brazil and thereby supports the development of prevention, interception, and control measures. Cases reported after 2013 were excluded due to possible delays in notification. Datasets by microregion, year, age group, gender, and clinical evolution were obtained and analysed. Additionally, datasets of clinical disorder and co-infection with HIV were produced for cutaneous and visceral leishmaniasis, respectively. Population data were acquired from the Brazilian Institute of Geography and Statistics (IBGE), and were supported by national census data from 2010 and official estimates for the other years (available at http://www.ibge.gov.br).

### Statistical analysis and geoprocessing

Statistical analyses were performed using the Epi Info™ software (version 6.04d, Centre for Disease Control and Prevention, www.cdc.gov/epiinfo). Categorical variables were analyzed using the chi-square test. Relative risk and 95% confidence intervals (*CI*s) were used to evaluate significant effects of each variable; if the 95% *CI*s did not contain unity, the effect was considered significant. A *P*-value of less than 0.05 was considered significant for all tests. Geographic information system techniques and spatial analysis tools were employed to determine the geographical distribution of cutaneous and visceral leishmaniasis, malaria, schistosomiasis, and acute Chagas disease in Brazil. The microregion was adopted as a unit of analysis in order to obtain improved accuracy concerning the differences among regions. Thematic maps were drawn in accordance with the rate per 100,000 inhabitants. Digital maps were obtained from the IBGE cartographic database in shape file (.shp), which were formatted and analysed using TerraView version 4.2, a public-access software provided by the National Institute for Space Research (www.dpi.inpe.br/terraview).

### Ethical considerations

This investigation was performed according to public domain data without the possibility of identifying subjects, thereby dispensing the need for approval by an institutional review board for human research.

## Results

In total, 64,567 cases of cutaneous and visceral leishmaniasis, malaria, schistosomiasis, and acute Chagas disease were recorded in the SINAN database, representing a rate of 20.15 cases per 100,000 inhabitants. The average age of the cases was 12.2 years (range 0–19 years old), There were more cases among males than females (40,239, 62.32% were male cases). Information about gender was missing for eight reported cases (two for schistosomiasis and six for cutaneous leishmaniasis [CL]).

The average age of cases was 12.2 years (range: 0–19 years). About a third (21,265, 32.93%) were aged 15–19 years, 18,456 (28.58%) were aged 10–14 years, and 13,299 (20.60%) were under 4 years of age. A lower predominance was documented in individuals aged 5–9 years (11,547; 17.88%). Overall, 0.62% of the reported cases were fatal.

**Fig. 2:**
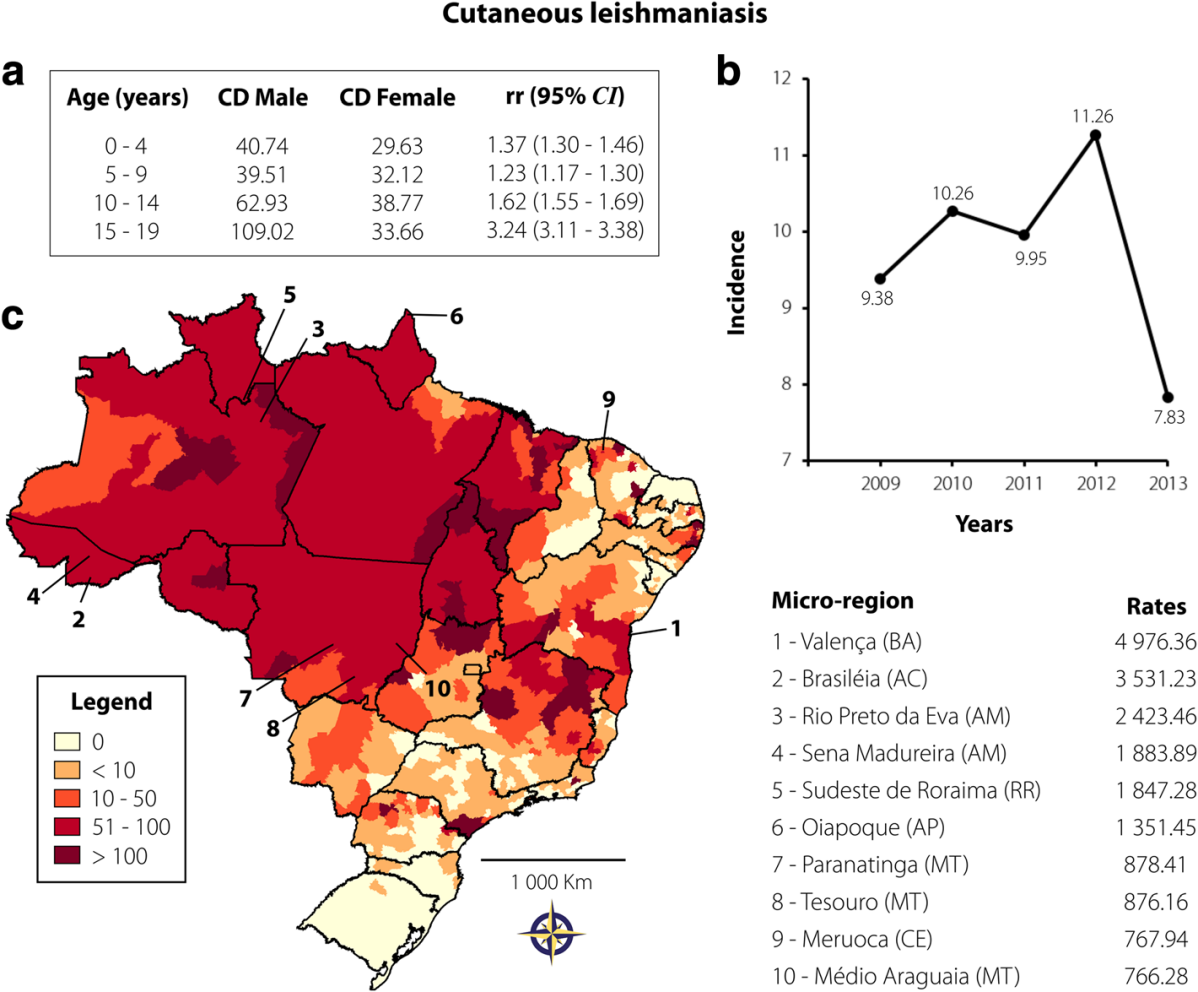
Cutaneous leishmaniasis transmission in Brazil from 2009 to 2013 in individuals aged 0–19 years. **a** Coefficient of detection (CD) and relative risk (rr), according to age and gender; **b** Rate of reported cases per 100,000 inhabitants according to year of notification; **c** Spatial distribution adopting the microregion as the unit of analysis. On the right are the 10 microregions that reported the highest case rates per 100,000 inhabitants

Cutaneous leishmaniasis was responsible for 31,178 of all reported cases (48.29%), with a rate of 9.73 cases per 100,000 inhabitants. This infection was substantially associated with male children and adolescents aged 15–19 years (see Fig. 2a). New reported cases were consistent throughout the period of the study, except for a slight decline in 2013 (see Fig. 2b). Of these 31,178 cases, 97% developed cutaneous disorders and 3% presented mucosal lesions. Cases of CL were homogeneously distributed in all regions of Brazil. The areas showing the highest rates are those located in the North and Central West, with up to 870 cases per 100,000 inhabitants. However, the microregion with the highest number of reported cases was located in the Northeast: Valença, reaching a peak of 4976.36 cases per 100,000 inhabitants. High numbers of cases were also reported in other microregions of the Northeast, such as Meruoca and Médio Araguaia. Few microregions reported new cases: Rio Grande do Norte, Rio Grande do Sul, and Santa Catarina (see Fig. 2c). The mortality rate of CL was 0.01%.

**Fig. 3:**
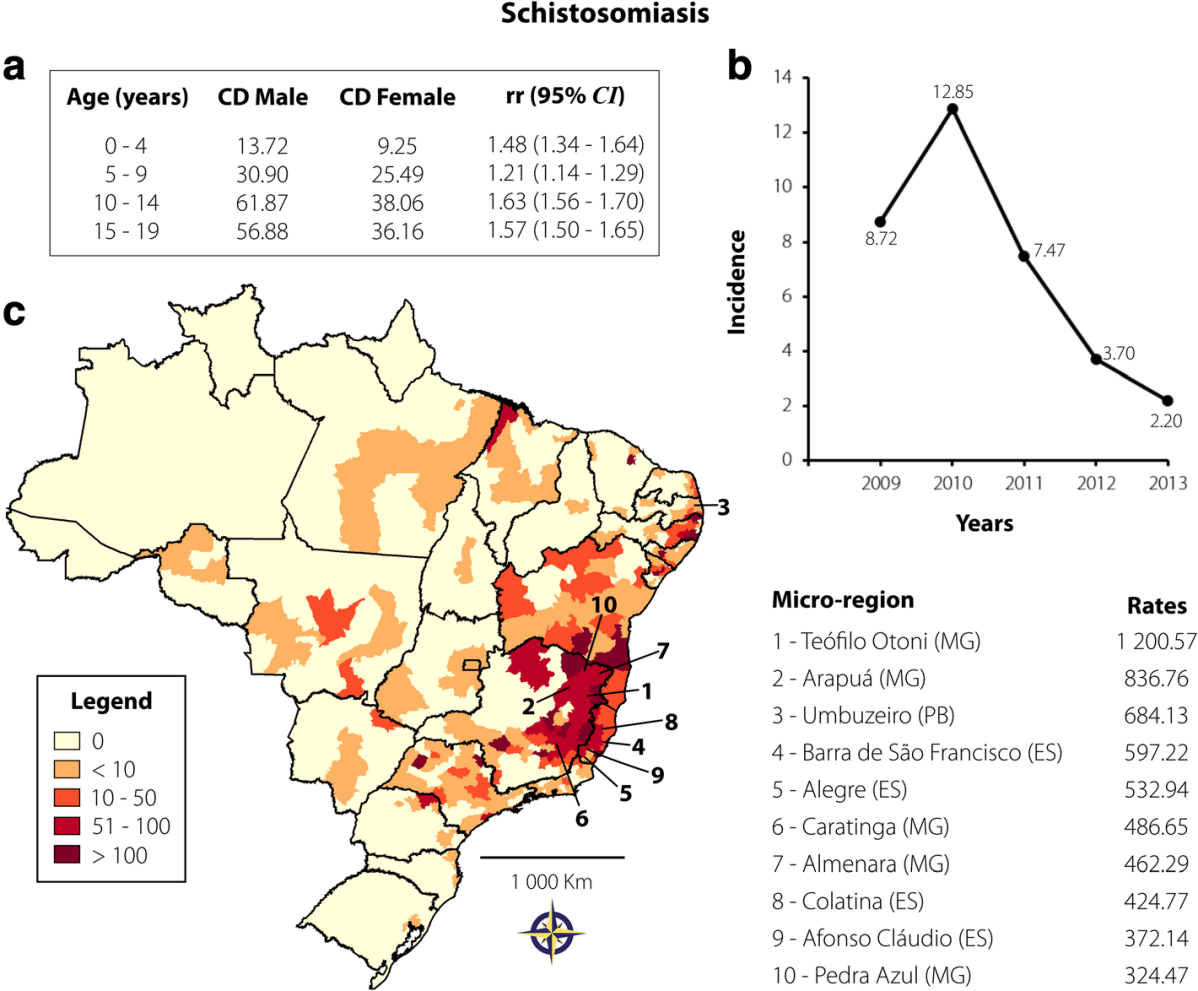
Schistosomiasis transmission in Brazil from 2009 to 2013 in individuals aged 0–19 years. **a** CD and rr, according to age and gender; **b** Rate of reported cases per 100,000 inhabitants according to year of notification; **c** Spatial distribution adopting the microregion as the unit of analysis. On the right are 10 microregions that reported the highest case rates per 100,000 inhabitants

Schistosomiasis presented the second most reported parasitic infection: 22,348 cases were reported (34.61%), representing a rate of 6.98 cases per 100,000 inhabitants. The highest predominance was recorded in individuals aged 10–14 (38.53%) and 15–19 (35.43%) years. The proportion of cases in all age groups was found to be significantly higher in males than in females (see Fig. 3a). A pronounced decrease in the notification of new cases has been recorded from 2010 to 2013 (see Fig. 3b). From the map in Fig. 3c, it can be observed that the highest incidences of schistosomiasis were recorded in the Northeast and Southeast regions of the country. They were mainly recorded in the Southeast, precisely the northeast of Minas Gerais and east of Espírito Santo states, following the route of the main river basins. Nine of the ten microregions with the highest incidences are located in these states. Only Umbuzeiro, with 684.13 cases per 100,000 inhabitants, is located in Paraíba state, in the Northeast region. Of the total cases of schistosomiasis, 0.05% led to death.

**Fig. 4:**
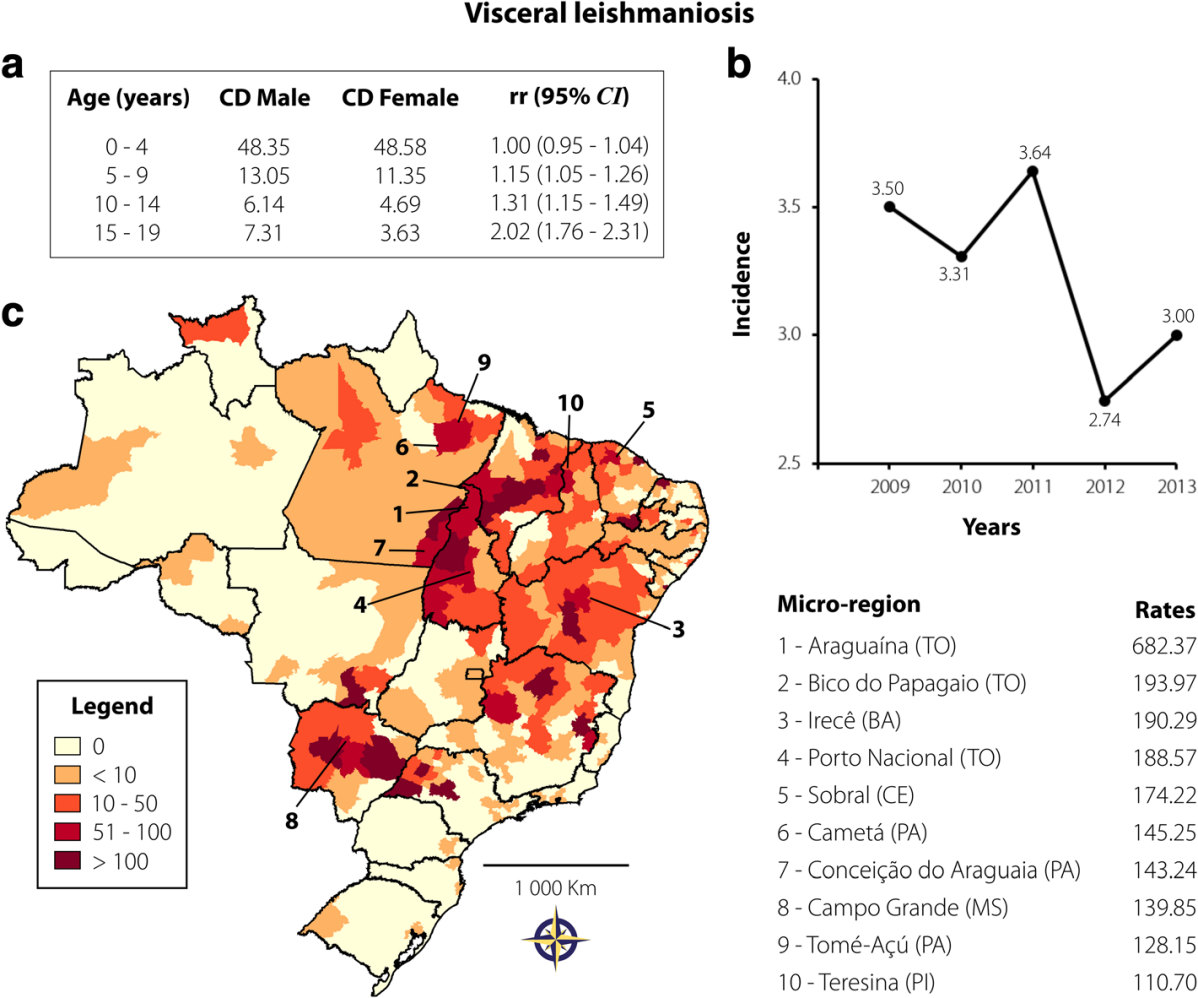
Visceral leishmaniasis transmission in Brazil from 2009 to 2013 in individuals aged 0–19 years. **a** CD and rr, according to age and gender; **b** Rate of reported cases per 100,000 inhabitants according to year of notification; **c** Spatial distribution adopting the microregion as the unit of analysis. On the right are 10 microregions that reported the highest case rates per 100,000 inhabitants

A total of 10,379 individuals (16.07%) with VL were identified, with a rate of 3.24 cases per 100,000 inhabitants. Of these, 6686 (64.42%) were children aged up to 4 years, 1829 (17.62%) were aged 5–9 years, 932 (8.98%) were aged 10–14 years, and 932 (8.98%) were aged 15–19 years. Regarding gender, the infection was significantly associated with males aged 15–19 (see Fig. 4a). Co-infection with HIV was reported in 7.14% of these individuals. The number of new cases during the period analysed was almost constant, with a slight reduction from 2012 to 2013 (see Fig. 4b). Figure 4c shows the areas with the highest incidence. Araguaína, Bico do Papagaio, Porto Nacional, Sobral, Cametá, Conceição do Araguaia, Tomé-Açú, and Teresina microregions presented more than 100 cases per 100,000 inhabitants. Other areas from Bahia (Irecê) and Mato Grosso do Sul (Campo Grande) were also affected, reporting 139.85 and 190.29 cases per 100,000 inhabitants, respectively. Of all the NTDs studied, VL had the highest death rate (3.72%).

**Fig. 5:**
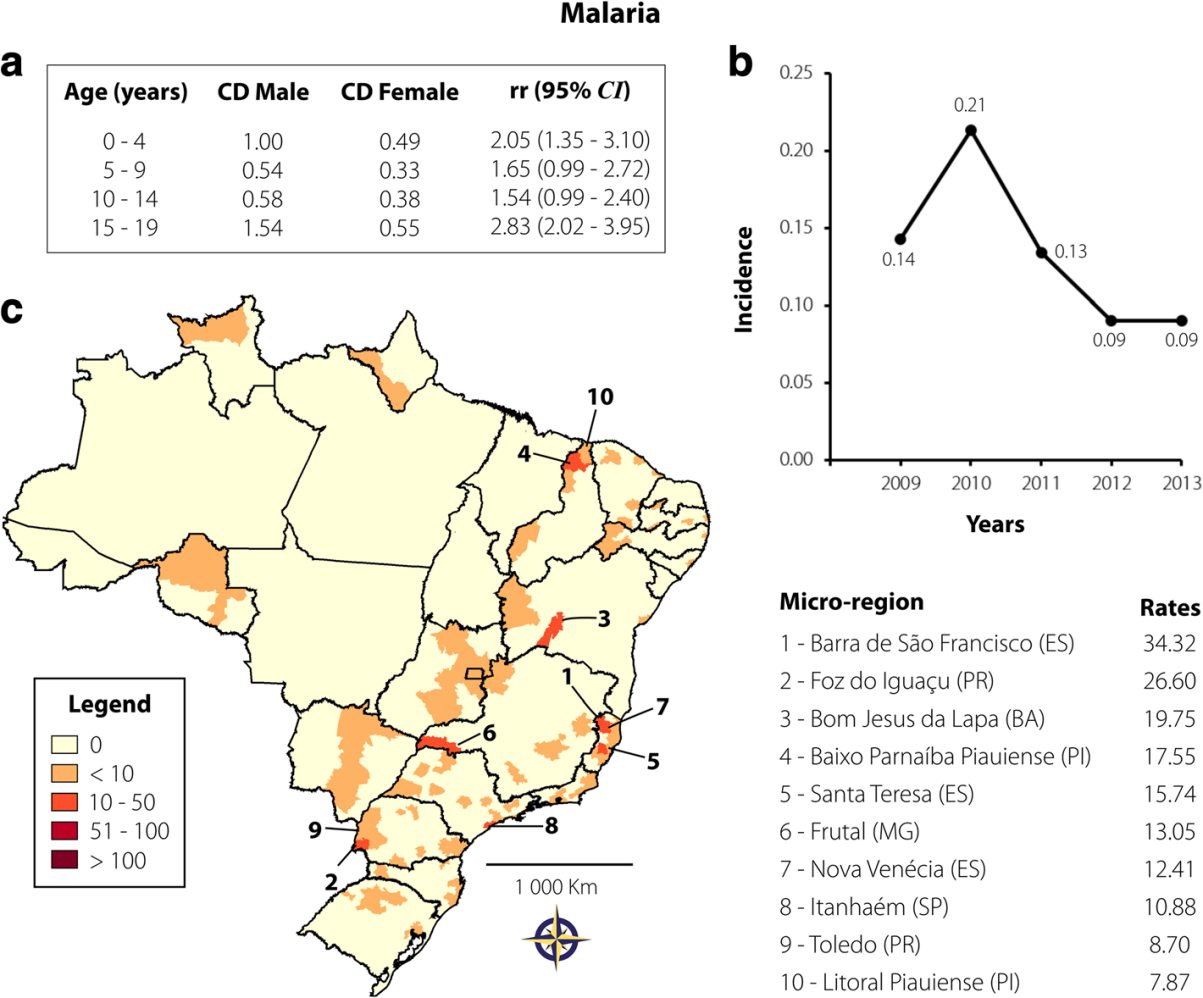
Malaria transmission in Brazil from 2009 to 2013 in individuals aged 0–19 years. **a** CD and rr, according to age and gender; **b** Rate of reported cases per 100,000 inhabitants according to year of notification; **c** Spatial distribution adopting the microregion as the unit of analysis. On the right are 10 microregions that reported the highest case rates per 100,000 inhabitants

Malaria was reported in 429 cases (0.66%), with a rate of 0.13 cases per 100,000 inhabitants. High incidence was observed in individuals aged 15–19 years (41.49%), followed by 10–14 years (19.35%), 4 years (16.08%), 5–9 years (15.15%), and up to 1 year (7.93%). The occurrence of the infection was higher in males only in the 0–4 and 15–19-year age groups (see Fig. 5a). As according to Fig. 5b, the number of reported cases have reduced from 2011 to 2013. The spatial distribution of malaria cases throughout the period analysed was heterogeneous, with a high concentration in Barra de São Francisco, Foz do Iguaçu, Bom Jesus da Lapa, Baixo Parnaíba Piauiense, and Santa Teresa microregions, which presented rates higher than 15 cases per 100,000 inhabitants (see Fig. 5c). No data about mortality from malaria were available.

**Fig. 6:**
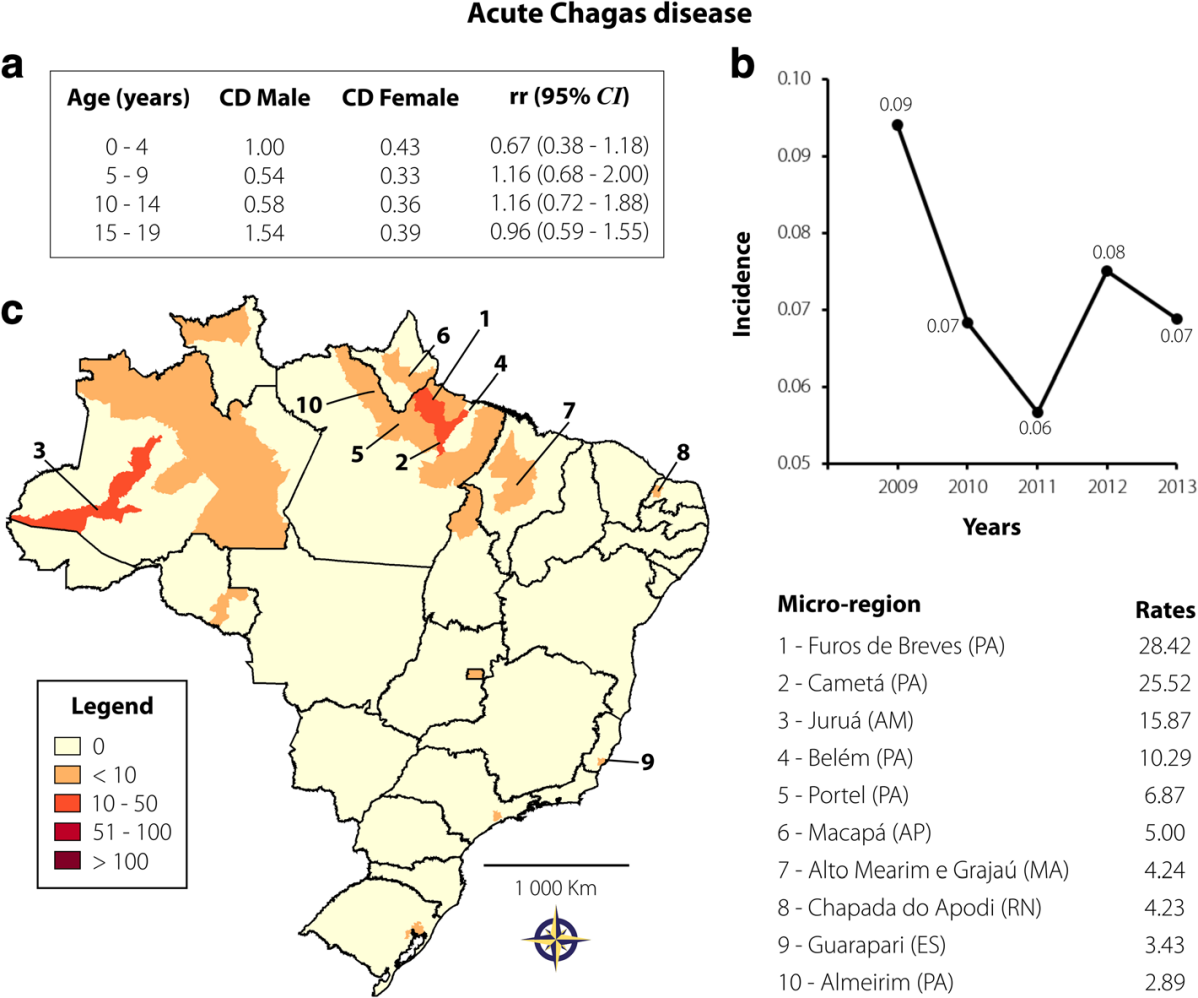
Acute Chagas disease transmission in Brazil from 2009 to 2013 in individuals aged 0–19 years. **a** CD and rr, according to age and gender; **b** Rate of reported cases per 100,000 inhabitants according to year of notification; **c** Spatial distribution adopting the microregion as the unit of analysis. On the right are 10 microregions that reported the highest case rates per 100,000 inhabitants

Acute Chagas disease presented the lowest number of reported cases: 233 reported cases (0.36%), representing a rate of 0.07 cases per 100,000 inhabitants. There were no gender differences with regards to age groups (see Fig. 6a) and a slight reduction in the number of new cases was observed from 2011 to 2013 (see Fig. 6b). The spatial distribution rates are depicted in Fig. 6c. Overall, a concentration of new cases in the North region is evident, more precisely in Pará, Amazonas, and Amapá states. Furo de Breves and Cametá, in Pará, showed a rate of higher than 25 cases per 100,000 inhabitants. Some areas of other Brazilian regions were also affected, varying from 3.43 to 4.24 cases per 100,000 inhabitants (Alto Mearim e Grajaú, Chapada do Apodi, Guarapari). Acute Chagas disease had the second highest mortality rate (1.72%).

## Discussion

The distribution of four NTDs in Brazil over a five-year period has been described in this study. In general, NTDs, such as malaria, acute Chagas disease, leishmaniasis (visceral and cutaneous), or schistosomiasis, impair lives in Brazil, affecting more than 20 per 100,000 inhabitants aged up to 19 years.

The highest number of reported cases is attributed to CL, followed by schistosomiasis, VL, malaria, and acute Chagas disease. Owing to the extensive geographic distribution of these diseases, particularly remarkable for CL, control efforts and elimination actions constitute a large-scale strategy. The adoption of the epidemiologic triad model as a preventive measure can interrupt the interaction among its constituent factors (host-antigen-environment) reducing the reporting of new cases. In line with this strategy, the Southern Cone Initiative successfully halted the transmission of Chagas disease by eliminating domestic populations of *T. infestans* and interrupting transfusional Chagas disease in vast areas of previously endemic regions [24].

Leishmaniasis is estimated to cause 1.6 million new cases worldwide each year, of which an estimated 1.1 million are cutaneous and 500,000 are visceral. However, of the 1.6 million estimated cases, 1 million remain unreported [20]. Since the late 1990s, Brazil has experienced an increase in the number of leishmaniasis cases, owing to the introduction of the parasite into suburban areas of large cities, resulting from the large-scale migration of people [20]. Indeed, a significant spatial distribution of CL in all Brazilian regions was identified in our study, constituting a widespread problem for public health. The vast majority of the reported cases occurred in the North region, more precisely in certain microregions of the Acre, Amazonas, Roraima, and Amapá states, with more than 11,946 cases per 100,000 inhabitants aged up to 19 years. Furthermore, clusters of microregions were identified with rates of over 750 reported cases per 100,000 in the states of Mato Grosso (Paranatinga and Tesouro microregions) and Ceará (Meruoca microregion). One particular microregion of southern Bahia caught the authors’ attention: Valença, characterized by approximately 5000 reported cases per 100,000 inhabitants aged up to 19 years. One village located in this microregion, Corte de Pedra, is recognized as one of the most important areas of *Leishmania braziliensis* transmission in Brazil. In general, CL in this region may be considered an occupational disease, since most of the infected individuals are members of the agricultural workforce, which means that healthy individuals are put in close contact with the wild cycle of the parasite, creating a high risk of exposure to infected phlebotomine bites. The village has experienced a sharp increase in the number of CL cases since 1983 [29, 30], leading to an outbreak in 1986 [31]. The last epidemiological study revealed an annual incidence of 8.1 cases per 1000 inhabitants and a prevalence of 14.9% [32].

Similarly, VL cases were identified in all Brazilian states, with greater concentrations in microregions located in the centre of the country. Araguaína (Tocantins state) had the highest rate: approximately 682 cases per 100,000 inhabitants aged up to 19 years. Our rate of reported cases is higher than the 578.39 cases (per 100,000) estimated in a cross-sectional study of children below 15 years of age, which was carried out in the municipality of Araguaína in 2007 [33]. The higher rate obtained in this study indicates an increase in the number of cases since the cross-sectional study was conducted. Cases of VL were also reported with a high burden (> 100 cases/100000 inhabitants aged up to 19 years) in other microregions, mainly in the North (Tocantins and Pará states); Northeast (Bahia, Ceará, and Piauí states); and Central-West regions (Mato Grosso do Sul state). Visceral leishmaniasis is potentially fatal if not treated promptly, mainly when associated with other risk factors, such as increased age, malnutrition, and immunosuppression. With respect to immunosuppression, it was observed that approximately 7% of VL cases were co-infected with HIV. According to Monge-Maillo and López-Vélez [34], VL and HIV co-infection has significant clinical, diagnostic, and epidemiological implications, favouring the reactivation of latent leishmaniasis, inducing a more severe manifestation of the disease, increasing the risk of relapse after treatment, and leading to a poorer therapeutic response.

Although commonly reported, CL showed low death rates compared with VL. If not treated in time, VL has a high mortality rate, with estimates varying between 80 and 100%. Even with treatment, fatality rates exceed 10% [35]. In Bangladesh, a study reported case-fatality rates of 6% and 8% among males and females below 15 years of age, respectively [35]. A high mortality rate was reported in Sergipe in Brazil from 1980 to 2013. During this period, 11.8% of deaths were attributed to VL [36]. In this study, however, a lower rate of death in individuals aged up to 19 years was found (3.72%).

Approximately 207 million people may have schistosomiasis worldwide, with an additional 779 million at risk of infection [37]. In Brazil, 2.5 million individuals are considered infected and 25 million are at risk of infection [38]. People become infected during contact with freshwater bodies infested with cercariae released by specific intermediate host snails when conducting recreational, domestic, and occupational activities. With regards to geographical distribution, the situation is different to that found for both cutaneous and visceral leishmaniasis. Even though a large proportion of the Brazilian states reported new cases of schistosomiasis, we observed a big concentration in Minas Gerais, Espírito Santo, and the coastal area of some Northeast states. The Téofilo Otoni microregion (Minas Gerais state) conferred the highest number of reported cases per 100,000 inhabitants aged up to 19 years. Indeed, a study carried out in Malacacheta, a municipality located in Téofilo Otoni, highlighted the high prevalence of infection by *S. mansoni* in schoolchildren, mainly in boys aged ≥11 years living in rural areas [39]. Similarly, a significant proportion of reported cases was found among boys aged 10–19 years. Exposure to leisure activities associated with river water may be related to the greater occurrence in this group. In this study, schistosomiasis presented low mortality rates. A study conducted in Brazil in 2007 revealed a 62.9% reduction in the mortality rate of schistosomiasis [40].

The past decade has seen the world make considerable progress in reducing malaria cases and deaths. However, most endemic areas are still far from achieving coverage with antimalaria programs. Globally, an estimated 198 million people have become infected with *Plasmodium* spp. and 584,000 people have died of malaria, with the heaviest burden in African countries, where children aged below 5 years account for 78% of all deaths [41]. Brazil contributed with a reduction in incidence of 76.8% between 2000 and 2014. Nevertheless, transmission remains dominant in the Amazon region, accounting for almost all reported cases in Brazil [42]. In contrast, our study did not show reported cases in the Amazonas, Acre, Tocantins, and Pará states, suggesting either successful prevention campaigns, underreported cases, or both. Although malaria prevalence has gradually been declining in Brazil [43, 44], we believe that the number of underreported cases in the North region is high, as some studies have described cases in children [45, 46] in this area. On the other hand, the spatial distribution of malaria has been found to be heterogeneous in other Brazilian regions. Cases were reported with either lesser or greater intensity in microregions of the Northeast, Southeast, and South regions. Indeed, microregions with more than 15 cases per 100,000 inhabitants aged up to 19 years are spatially far from one another, suggesting that control campaigns against *Plasmodium* spp. should be mandatory nationwide.

Between 2001 and 2006, 2476 cases of acute Chagas disease (64.7% vectorial, 0.7% transfusional, 0.3% transplacental, and 34.3% of unknown transmission route) were reported in Brazil [47]. However, as previously mentioned, the Southern Cone Initiative has changed the epidemiological profile of the disease, as the country has received international certification for the interruption of vectorial transmission by *T. infestans* in 2006. In fact, a study carried out in Pernambuco state showed a significant decrease in new cases starting in 2006 [25]. Because of the decrease in vectorial transmission in Brazil, attention should be directed to the new cases that occur through contaminated food and beverages in endemic areas [48]. In this study, a high burden of acute Chagas disease in the states of the Amazon Basin, mainly Amazonas and Pará, was found. Oral transmission of this disease in the Amazon region has been reported since the 1960s and our data are in agreement with previous studies [49, 50]. Acute Chagas disease, the NTD with the lowest number of reported cases, was shown to be responsible for 1.72% of deaths in inhabitants aged up to 19 years. The high death rate for Chagas disease is a peculiar characteristic of endemic countries in Latin America. However, deaths attributed to Chagas disease are more common in individuals aged over 40 years, who had probably acquired the infection earlier in their lives [36, 51]. Acute Chagas diasease was also the most common cause of mortality in the general population in a study that assessed reported cases from 2000 to 2011, revealing a significant public health problem in Brazil [52].

## Conclusions

The number of cases and the lethality rate described show that the four NTDs examined in this study still represent a serious public health problem in Brazil. This points to the need to encourage new research and the reformulation of social, economic, and public health policies aimed at ensuring better health and living conditions for all individuals, especially those among the populations considered vulnerable, as is the case of the young. The analysis of the geographical distribution of these NTDs enables the identification of priority areas for the development and intensification of control and elimination initiatives.

## Additional files

Additional file 1: Multilingual abstracts in the five official working languages of the United Nations. (PDF 667 kb)

Additional file 2: Neglected diseases: the strategies of the Brazilian Ministry of Health. (PDF 100 kb)

## Abbreviations

*CI*: Confidence interval
CL: Cutaneous leishmaniasis
IBGE: Brazilian Institute of Geography and Statistics
NTD: Neglected tropical disease
SINAN: Health Notification Aggravation Information System
VL: Visceral leishmaniasis
WHO: World Health Organization
