# Supplementary material for: Neglected tropical diseases in Brazilian children and adolescents: data analysis from 2009 to 2013
Source: Infect Dis Poverty. 2017 Nov 3;6:154. doi: 10.1186/s40249-017-0369-0 (PMC5668976; doi:10.1186/s40249-017-0369-0)
Supplement: Supplementary file 1 — Multilingual abstracts in the five official working languages of the United Nations. (PDF 667 kb) [file 40249_2017_369_MOESM1_ESM.pdf]

Translation of the abstract into the five official working languages of the United Nations

أمراض المناطق المدارية المهملة في الأطفال والمراهقين البرازيليين: تحليل البيانات من 2009 إلى 2013

إدواردو برانداو، سيباستيان روميرو، ماريا ألمريس لوبيس دا سيلفا، فريد لوتشيانو نيفيس سانتوس

ملخص

الخلفية: تسود الأمراض المدارية المهملة في ظروف الفقر وتسهم في الإبقاء على عدم المساواة الاجتماعية. ومن بين الأمراض المدارية المهملة التي تعطيها وزارة الصحة البرازيلية الأولوية، تتطلب أربعة أمراض طفيلية معدية إخطاراً إلزامياً: مرض شاغاس الحاد وداء الليشمانيات والملاريا وداء البلهارسيا. إن البيانات المتعلقة بسلوك أمراض المناطق المدارية المهملة هذه بين السكان الشباب محدودة حالياً. وتسعى هذه الدراسة إلى تحليل الجوانب الوبائية لهذه الإصابات الطفيلية لدى الأطفال والمراهقين في البرازيل.

الطرق: تم إجراء دراسة استعادية استكشافية بيئية. وتم إجراء تحليل مكاني للحالات المبلغ عنها بين أعوام 2009 و 2013 لدى الأفراد الذين تتراوح أعمارهم بين 0 و 19 عاماً والذين تم إخطارهم من خلال نظام معلومات تفاقم الإشعار الصحي (SINAN).

النتائج: في المجموع، سجلت 64 567 حالة من داء الليشمانيات الجلدي والحشوي والملاريا والبلهارسيا ومرض شاغاس الحاد في قاعدة معلومات تفاقم الإشعار الصحي (SINAN)، يمثلون نسبة 20.15 حالة لكل 100000 من السكان. كان متوسط العمر من الحالات 12.2 سنة و 62.32 % من الذكور. وتم تسجيل أربع مائة وثلاث وفيات مرتبطة بهذه الطفيليات المذكورة إلزامياً، مما يشير إلى معدل حالات وفيات 0.62 %. سجل داء الليشمانيات الحشوي وداء شاغاس الحاد أعلى معدلات للإماتة. وقد لوحظ توزيع مكاني غير متجانس للطفيليات المدروسة.

الاستنتاجات: يبين عدد الحالات ومعدل الإماتة الموصوف في هذه الدراسة أن هذه الأمراض لا تزال تمثل مشكلة خطيرة بالنسبة للصحة العامة في البرازيل. وهذا يشير إلى الحاجة لتشجيع الأبحاث الجديدة وإعادة صياغة سياسات اجتماعية واقتصادية، وسياسات صحة عامة تهدف إلى ضمان ظروف صحية ومعيشية أفضل لجميع الأفراد، لا سيما بين السكان الذين يعتبرون معرضون للخطر، كما هو الحال بالنسبة الشباب.

Translated from English version into Arabic by Free bird, through

TRANSLATORS
WITHOUT BORDERS

2009-2013 年巴西儿童和青少年被忽视的热带病的数据分析

Eduardo Brandão, Sebastián Romero, Maria Almerice Lopes da Silva, Fred Luciano Neves Santos

摘要

引言：被忽视的热带病（NTDs）在贫困区普遍流行，不利于维护社会平等。巴西卫生部优先解决的 NTDs 中，4 种寄生虫感染需要强制报告：急性美洲锥虫病、利什曼病、疟疾和血吸虫病。目前，这些 NTDs 在年轻人群的数据有限。本研究旨在分析巴西儿童和青少年寄生虫感染的流行病学特征。

方法：我们开展了回顾性探索性生态学研究，通过健康通报信息系统（SINAN）收集 2009-2013 年 0-19 岁人群的病例，并进行空间分析。

结果：在 SINAN 数据库中共记录了 64 567 例皮肤和内脏利什曼病、疟疾、血吸虫病和急性美洲锥虫病例。发病率为 20.15/10 万。平均年龄为 12.2 岁，男性占 62.32%。强制报告的寄生虫相关死亡病例数为 403 例，其死亡率为 0.62%。内脏利什曼病和急性美洲锥虫病的致死

率最高。本研究纳入的寄生虫呈异质性空间分布。
结论：本研究中描述的病例数和致死率表明，这些疾病仍然是巴西公共卫生的严重问题。研究表明，需要鼓励创新研究，重新制定社会、经济和公共卫生政策，以确保所有个体特别是脆弱人群和年轻人享有更好的健康和生活条件。

Translated from English version into Chinese by Jin Chen, edited by Pin Yang

CDC CHINESE CENTER FOR DISEASE CONTROL AND PREVENTION
NATIONAL INSTITUTE OF PARASITIC DISEASES

Les maladies tropicales négligées parmi les enfants et adolescents brésiliens : analyse des données de 2009 à 2013

Eduardo Brandão, Sebastián Romero, Maria Almerice Lopes da Silva, Fred Luciano Neves Santos

Résumé

Contexte : Les maladies tropicales négligées (MTN) prévalent dans les conditions de pauvreté et contribuent à la perpétuation des inégalités sociales. Parmi les MTN dont le Ministère de la Santé brésilien a fait sa priorité, quatre parasitoses sont à déclaration obligatoire : maladie de Chagas aiguë, leishmaniose, paludisme et schistosomiase. Les données concernant le comportement de ces MTN dans les tranches d'âge les plus jeunes sont actuellement limitées. La présente étude analyse les aspects économiques de ces parasitoses chez les enfants et adolescents brésiliens.
Méthodes : Une étude écologique exploratoire rétrospective a été menée. Une analyse spatiale des cas rapportés entre 2009 et 2013 chez les sujets âgés de 0 à 19 ans rapportés au système de surveillance national (SINAN) a été réalisée.
Résultats : Au total, 64 567 cas de leishmaniose cutanée et viscérale, de paludisme, de schistosomiase et de maladie de Chagas aiguë ont été enregistrés dans la base de données SINAN, ce qui représente 20,15 cas par 100 000 habitants. L'âge moyen des cas était de 12,2 ans et 62,32 % étaient de sexe masculin. Quatre cent trois décès imputables à ces parasitoses à déclaration obligatoire ont été enregistrés, ce qui indique un taux de létalité de 0,62 %. La leishmaniose viscérale et la maladie de Chagas aiguë étaient les plus létales. Nous avons noté une distribution spatiale hétérogène des parasites étudiés.
Conclusions : Le nombre de cas et le taux de létalité décrits dans cette étude montrent que ces maladies posent toujours un sérieux problème de santé publique au Brésil. Cela met en lumière la nécessité d'encourager de nouvelles recherches et de reformuler les politiques sociales, économiques et de santé publique afin d'assurer une meilleure santé et de meilleures conditions de travail pour tous, en particulier les catégories de la population considérées comme vulnérables, comme les plus jeunes.

Translated from English version into French by Suzanne Assenat, through

TRANSLATORS
WITHOUT BORDERS

подростков: анализ данных с 2009 по 2013 гг.

Эдуардо Брандау, Себастьян Ромеро, Мария Алмерис Лопеш да Сильва, Фред Лучано, Невес Сантос

Аннотация

Справочная информация: Забытые тропические болезни (ЗТБ) преобладают в условиях нищеты и способствуют сохранению социального неравенства. Из общего числа ЗТБ, определённых в качестве приоритетов Министерством здравоохранения Бразилии, обязательное уведомление требуется по четырем паразитарным инфекциям: по острой болезни Шагаса, по лейшманиозу, по малярии и шистосомозу. В настоящее время в наличии имеются лишь ограниченные данные о поведении указанных ЗТБ среди молодежи. Целью данного исследования является анализ эпидемиологических аспектов упомянутых паразитических инфекций у детей и подростков в Бразилии.

Методы: Было проведено ретроспективное предварительное экологическое исследование. Был выполнен пространственный анализ клинических случаев, зарегистрированных в период с 2009 по 2013 годы среди лиц в возрасте от 0 до 19 лет, уведомлённых посредством информационной системы уведомления об ухудшении состояния здоровья (SINAN).

Результаты: В системе уведомления SINAN было зарегистрировано в целом 64 567 клинических случаев кожного и висцерального лейшманиоза, малярии, шистосомоза и острой болезни Шагаса, что соответствует 20,15 случаям на каждые 100 000 человек населения. Средний возраст зарегистрированных случаев составил 12,2 года и 62,32% от общего числа включал население мужского пола. Связь с указанными паразитами, подлежащими обязательному уведомлению, была установлена в четырехста трёх фатальных исходах, что свидетельствует о показателе смертности от данного заболевания в 0,62%. Самые высокие показатели смертности были обнаружены при висцеральном лейшманиозе и острой болезни Шагаса. Было отмечено неоднородное пространственное распределение исследуемых паразитов.

Выводы: Число случаев, а также уровень смертности, описанные в данном исследовании указывают на то, что означенные болезни до сих пор представляют собой серьёзную проблему для здоровья населения Бразилии. Это указывает на необходимость поощрения новых исследований и пересмотра как социальной и экономической политики, так и политики по вопросам здравоохранения, направляя их на обеспечение улучшения состояния здоровья и условий жизни для всех людей, особенно среди уязвимых групп населения, таких как молодёжь.

Translated from English version into Russian by Liudmila Tomanek (nee Volynets), through

TRANSLATORS WITHOUT BORDERS

Enfermedades tropicales desatendidas en niños brasileños y adolescentes: Análisis de datos desde 2009 a 2013

Eduardo Brandão, Sebastián Romero, Maria Almerice Lopes da Silva, Fred Luciano Neves Santos

Resumen

Introducción: Las enfermedades tropicales desatendidas (ETD) prevalecen en condiciones de pobreza y contribuyen al mantenimiento de la inequidad social. Fuera de las ETD priorizadas por el Ministerio de Salud brasileño, cuatro infecciones parasíticas requieren notificación obligatoria: la enfermedad de Chagas aguda, la leishmaniasis, la malaria y la esquistosomiasis. Datos sobre la conducta de estas ETD sobre la población joven actualmente son limitados. Este estudio busca analizar los aspectos epidemiológicos de estas infecciones parasíticas en niños y adolescentes en Brasil.

Metodología: Se condujo un estudio ecológico-exploratorio-retrospectivo. Se realizó un análisis espacial de los casos reportados entre 2009 y 2013 de personas de entre 0 y 19 años de edad que fueron notificados a través del Sistema de Información sobre Enfermedades de Notificación Obligatoria (SINAN).

Resultados: En total, 64 567 casos de leishmaniasis visceral y cutánea, malaria, esquistosomiasis y enfermedad de Chagas aguda fueron registrados en la base de datos del SINAN, lo que representa una proporción de 20.15 casos cada 100 000 habitantes. La edad promedio de los casos fue de 12.2 años y 62.32% fueron masculinos. Se registraron cuatro cientos y tres muertes relacionadas con estos parásitos de notificación obligatoria, lo que indica una tasa de letalidad de 0.62%. La leishmaniasis y la enfermedad de Chagas aguda tuvieron la mayor tasa de letalidad. Se observó una distribución espacial heterogénea de los parásitos estudiados.

Conclusión: El número de casos y la tasa de letalidad descrito en este estudio muestran que estas enfermedades representan todavía un serio problema para la salud pública en Brasil. Esto indica la necesidad de fomentar investigaciones nuevas y la reformulación de las políticas sociales, económicas, y de salud pública con el objetivo de asegurar una mejor salud y condiciones de vida para todas las personas, especialmente aquellas en poblaciones que se consideran vulnerables, como es el caso de las de los jóvenes.

Translated from English version into Spanish by Franco Flesia, through

TRANSLATORS
WITHOUT BORDERS
