# Supplementary material for: Neglected tropical diseases in Brazilian children and adolescents: data analysis from 2009 to 2013
Source: Infect Dis Poverty. 2017 Nov 3;6:154. doi: 10.1186/s40249-017-0369-0 (PMC5668976; doi:10.1186/s40249-017-0369-0)
Supplement: Supplementary file 2 — Neglected diseases: the strategies of the Brazilian Ministry of Health. (PDF 100 kb) [file 40249_2017_369_MOESM2_ESM.pdf]

Rev Saúde Pública 2010;44(1):200-2

Departamento de Ciência e Tecnologia, Secretaria de Ciência, Tecnologia e Insumos Estratégicos, Ministério da Saúde

Doenças negligenciadas: estratégias do Ministério da Saúde

Neglected diseases: the strategies of the Brazilian Ministry of Health

Correspondência | Correspondence:
Decit – Departamento de Ciência e Tecnologia do Ministério da Saúde
Esplanada dos Ministérios
Bloco G sala 845
70058-900 Brasília, DF, Brasil

Texto de difusão técnico-científica do Ministério de Saúde.

Doenças negligenciadas são doenças que não só prevalecem em condições de pobreza, mas também contribuem para a manutenção do quadro de desigualdade, já que representam forte entrave ao desenvolvimento dos países. Como exemplos de doenças negligenciadas, podemos citar: dengue, doença de Chagas, esquistossomose, hanseníase, leishmaniose, malária, tuberculose, entre outras. Segundo dados da Organização Mundial de Saúde (OMS), mais de um bilhão de pessoas estão infectadas com uma ou mais doenças negligenciadas, o que representa um sexto da população mundial.

Embora exista financiamento para pesquisas relacionadas às doenças negligenciadas, o conhecimento produzido não se reverte em avanços terapêuticos, como, por exemplo, novos fármacos, métodos diagnósticos e vacinas. Uma das razões para esse quadro é o baixo interesse da indústria farmacêutica nesse tema, justificado pelo reduzido potencial de retorno lucrativo para a indústria, uma vez que a população atingida é de baixa renda e presente, em sua maioria, nos países em desenvolvimento.

DEFINIÇÃO DE PRIORIDADES

O Ministério da Saúde (MS) orienta seus investimentos em pesquisa pela Agenda Nacional de Prioridades de Pesquisa em Saúde (ANPPS),[a] aprovada na II Conferência Nacional de Ciência, Tecnologia e Inovação em Saúde, em 2004. Organizada em 24 sub-agendas, a Agenda também possui diversas linhas de pesquisa relacionadas às doenças negligenciadas, principalmente na sub-agenda de doenças transmissíveis.

Para determinar temas de pesquisa que comporão os editais públicos e em consonância com as necessidades brasileiras, são realizadas oficinas de prioridades com a participação de gestores, pesquisadores e profissionais da área de saúde. Essas oficinas têm por objetivo nortear as chamadas públicas realizadas pelo Ministério da Saúde em parceria com o Ministério da Ciência e Tecnologia (MCT), Ministério da Educação, fundações de amparo à pesquisa e secretarias estaduais de saúde e de ciência e tecnologia, entre outros parceiros.

FINANCIAMENTO EM DOENÇAS NEGLIGENCIADAS

As ações iniciais do Ministério da Saúde com relação às doenças negligenciadas foram lançadas em 2003, com o primeiro edital temático em tuberculose, seguido pelos editais de dengue (2004) e hanseníase (2005) – todos editais nacionais que seguiram definições das oficinas de prioridades realizadas em Brasília, DF.

Como desencadeamento desse processo, em 2006, foi realizada a primeira oficina de prioridades em doenças negligenciadas e iniciado o Programa de Pesquisa e Desenvolvimento em Doenças Negligenciadas no Brasil, no âmbito da parceria do MS com o MCT. Nova oficina de prioridades em doenças negligenciadas foi realizada em 2008, visando ao lançamento de edital temático na área. A Secretaria de Vigilância em Saúde do MS é também parceira desse programa, contribuindo tecnicamente para o processo de definição de prioridades na área e para a avaliação dos resultados das pesquisas financiadas.

Por meio de dados epidemiológicos, demográficos e o impacto da doença, foram definidas, entre as doenças consideradas negligenciadas, sete prioridades de atuação que compõem o programa em doenças negligenciadas: dengue, doença de Chagas, leishmaniose, hanseníase, malária, esquistossomose e tuberculose.

[a] A agenda está disponível para consulta em: http://bvsms.saude.gov.br/bvs/publicacoes/AGENDA_PORTUGUES_MONTADO.pdf

Por meio desse programa, já foram lançados dois editais temáticos que financiaram 140 projetos com um investimento total de R$ 39 milhões.

Além dos editais nacionais, o Departamento de Ciência e Tecnologia (Decit), da Secretaria de Ciência, Tecnologia e Insumos Estratégicos (SCTIE), também possui uma modalidade de fomento descentralizado – o Programa de Pesquisa Para o SUS (PPSUS) – que promove o financiamento de projetos de pesquisa em saúde em todos os estados brasileiros. Esse programa estimula o investimento das fundações de amparo à pesquisa estaduais, secretarias estaduais de Saúde, assim como secretarias de ciência e tecnologia estaduais e suas participações como gestores do programa. Dessa forma, além dos projetos financiados pelos editais temáticos nacionais, outros projetos na área de doenças negligenciadas foram financiados por editais do PPSUS. De 2003 a 2008, por exemplo, foram 203 projetos financiados, totalizando investimentos da ordem de R$ 10,6 milhões.

TRABALHO EM REDE

Com o intuito de fortalecer e qualificar ações em temas de pesquisa considerados prioritários para o SUS, o MS tem investido na organização de redes de pesquisa eficazes para a indução ao desenvolvimento de uma dada área de pesquisa, bem como para o fortalecimento da capacidade instalada nas instituições de pesquisa integrantes. Assim, em 2009, como parte das iniciativas voltadas para a priorização da pesquisa em doenças negligenciadas no Brasil, o MS (por meio do Decit) em parceria com o MCT (por meio do Conselho Nacional de Desenvolvimento Científico e Tecnológico – CNPq/MCT) e as fundações de amparo à pesquisa dos estados do Amazonas, Maranhão, Mato Grosso, Minas Gerais, Pará, Rio de Janeiro e São Paulo constituíram uma rede para fomentar pesquisas sobre malária, uma das doenças de maior ocorrência na região da Amazônia Legal.

A Rede Malária, que teve aporte financeiro inicial de R$ 15,4 milhões para o desenvolvimento de projetos em rede no período de três anos, tem por objetivo agregar diferentes competências de regiões distintas do País para o enfrentamento da malária. O intuito é estimular o intercâmbio entre instituições que concentram competências, a interação entre pesquisadores em toda a Amazônia Legal, o uso otimizado de recursos e o compartilhamento de infra-estrutura para a pesquisa, principalmente de equipamentos de custo elevado, usualmente inacessíveis quando solicitados individualmente.

Outra iniciativa, em 2009, foi o lançamento do edital de seleção de propostas para a implantação de uma rede inter-regional e interdisciplinar de pesquisas em dengue, como Subprograma Temático do Programa de Apoio aos Núcleos de Excelência (Pronex – Rede Dengue), no valor global de R$ 22,7 milhões. O edital é resultado de uma parceria entre o MS, o MCT e fundações de amparo à pesquisa de 19 estados e do Distrito Federal (Tabela).

A expectativa é que a criação dessas redes mobilize grupos científicos de destaque no País dedicados à temática de doenças negligenciadas, estimulando o intercâmbio e a cooperação de modo que suas distintas competências se articulem e se complementem. Assim, novos conhecimentos sobre os diferentes aspectos – biológicos, clínicos, sociais entre outros – da malária e da dengue serão mais rapidamente produzidos, contribuindo para o combate a essas doenças, tanto no que concerne aos indivíduos doentes ou expostos, quanto ao que tange às coletividades e suas condições de vida.

INSTITUTOS NACIONAIS DE CIÊNCIA E TECNOLOGIA

Também no intuito de agregar grupos de pesquisa e apoiar atividades de pesquisa científica, tecnológica e de inovação, em 2008, foram selecionados projetos para formação e consolidação de institutos nacionais de ciência e tecnologia (INCT), em uma iniciativa do MS, MCT, Coordenação de Aperfeiçoamento de Pessoal de Nível Superior (CAPES), Banco Nacional de Desenvolvimento Econômico e Social (BNDES) e das FAP do Amazonas, Minas Gerais, Pará, Rio de Janeiro, São Paulo e Santa Catarina.

Entre os INCT que tiveram recursos do MS, existem alguns com grande potencial de contribuição para a temática de doenças negligenciadas e o combate às doenças: INCT em Tuberculose, INCT de Gestão da Inovação em Doenças Negligenciadas, INCT de Biotecnologia Estrutural e Química Medicinal em Doenças Infecciosas e INCT de Vacinas.

Tabela. Grandes editais temáticos na área de doenças negligenciadas.

Ano	Edital	Recursos
2003	Rede Tuberculose	R$ 1,9 milhões
2004	Dengue	R$ 945 mil
2005	Hanseníase	R$ 2,5 milhões
2006	Doenças negligenciadas[a]	R$ 17 milhões
2008	Doenças negligenciadas	R$ 22 milhões
2009	Rede Malária	R$ 15,4 milhões
2009	Rede Dengue	R$ 22,7 milhões

[a]Diferentemente do edital de 2008, o edital de doenças negligenciadas de 2006 não incluiu esquistossomose.

DA TEORIA À PRÁTICA

Com o incremento de investimentos em doenças negligenciadas e de publicações na área, surgiu a demanda de transformar os conhecimentos produzidos em produtos para a população acometida por doenças negligenciadas. Com esse objetivo, o Ministério da Saúde, e a Fundação Oswaldo Cruz (Fiocruz), realizaram, em 2008, um encontro com representantes de diversas instituições e países, para definir estratégia de pesquisa translacional em doenças negligenciadas, de forma a transformar resultados de pesquisas em aplicações médicas para atender à população brasileira. A partir do encontro, foi elaborado um documento, entregue ao Ministro da Saúde José Gomes Temporão, visando à criação efetiva do Programa Translacional do Brasil em Doenças Negligenciadas.
